# Determining levels of cryptic diversity within the endemic frog genera, *Indirana* and *Walkerana*, of the Western Ghats, India

**DOI:** 10.1371/journal.pone.0237431

**Published:** 2020-09-02

**Authors:** Vijay Ramesh, S. P. Vijayakumar, Trisha Gopalakrishna, Aditi Jayarajan, Kartik Shanker

**Affiliations:** 1 Department of Ecology, Evolution & Environmental Biology, Columbia University, New York, NY, United States of America; 2 Centre for Ecological Sciences, Indian Institute of Science, Bangalore, Karnataka, India; 3 School of Geography and the Environment, University of Oxford, Oxford, United Kingdom; Centre for Cellular and Molecular Biology, INDIA

## Abstract

A large number of species in the tropics are awaiting discovery, many due to their cryptic morphology ie. lack of discernable morphological difference. We explored the presence of cryptic lineages within the frog genera, *Indirana* and *Walkerana*, which are endemic to the Western Ghats of Peninsular India. By reconstructing a phylogeny using 5 genes and robust geographic sampling, we delimited 19 lineages along a population—species continuum, using multiple criteria including haplotype clusters, genetic distance, morphological distinctness, and geographical separation. Of these 19 lineages, 14 belonged to the genus *Indirana* and 5 to the genus *Walkerana*. Divergence dating analyses revealed that the clade comprising *Indirana* and *Walkerana* began diversifying around 71 mya and the most recent common ancestor of *Indirana* and *Walkerana* split around 43 mya. We tested for the presence of cryptic lineages by examining the relationship between genetic and morphological divergence among related pairs within a pool of 15 lineages. The pairs showed strong morphological conservatism across varying levels of genetic divergence. Our results highlight the prevalence of morphologically cryptic lineages in these ancient endemic clades of the Western Ghats. This emphasizes the significance of other axes, such as geography, in species delimitation. With increasing threats to amphibian habitats, it is imperative that cryptic lineages are identified so that appropriate conservation measures can be implemented.

## Introduction

Species that are morphologically similar, but are genetically divergent, are termed cryptic species [1, 2]. Identifying and delimiting such species is a key challenge in evolutionary biology. Cryptic species accumulate genetic differences and diverge over time, but they exhibit limited to no differences in morphology. This lack of morphological differentiation is hypothesized to be a result of recent divergence events, which might not be sufficient for distinct morphological characters to evolve or could be a result of selection for morphological stasis [3–5]. Often, a cryptic species remains indistinguishable from related species and maintains reproductive isolation through mechanisms that cannot be discerned or detected [6]. In cases where genetic divergence is accompanied by phenotypic divergence, species delimitation is straightforward. However, in the case of cryptic taxa, we need multiple lines of evidence to provide a robust basis for distinguishing species.

With advances in genomic methods and wider geographic sampling, there is a significant increase in the number of cryptic species being reported [2]. In fact, cryptic species have been identified across all major branches of life, highlighting their importance [5, 7]. They form a large proportion of diversity throughout the tropics and across taxonomic groups [1, 8, 9]; they have been detected in a variety of groups ranging from nematodes to large mammals such as giraffes and orangutans [10–12]. In addition, they are a challenge for conservation since many endangered species might consist of multiple cryptic species that may be rarer than previously thought [2]. Considering their prevalence and the implications for both conservation as well as evolutionary biology, a rigorous approach to identifying cryptic species is essential [1, 13].

Struck et al. [5] proposed a conceptual framework to delimit cryptic species that suggests accounting for levels of genetic divergence and phenotypic differences. While a large fraction of biodiversity is hypothesized and reported to be cryptic, inconsistency in methodological approaches in the identification of such taxa has led to further confusion. For example, of the 606 studies surveyed in Struck et al. [5], only ~ 40% of them used both genetic and morphological/phenotypic data, despite claiming the presence of cryptic diversity. Thus, we need to consider multiple lines of evidence such as geography, morphology, and genetics within a robust statistical framework to delimit cryptic diversity. As the tropics and other parts of the planet undergo drastic changes and massive conversion of suitable habitat into agricultural land, forest plantations and industrial zones, it is imperative that we identify and conserve these cryptic lineages before they decline or are extirpated [14, 15].

One such tropical region is the Western Ghats, a biodiversity hotspot that is home to numerous endemic species of amphibians [16]. The complex topography of this region encompasses major geographic gaps which have been shown to act as biogeographic barriers for a range of taxa [17, 18]. This could create potential opportunities for non-adaptive diversification resulting in cryptic lineages. *Indirana*, an endemic genus in the Western Ghats, belongs to one of the ancient clades of frogs that originated in the Gondwana and diversified in-situ [19]. Recent studies have suggested the existence of genetically divergent lineages, including new species and as well as a new genus–*Walkerana*–within this clade [20–23]. An earlier study by Nair et al. [24] brought to light the occurrence of cryptic diversity in the genus *Indirana* in the Western Ghats; however, this has not been formally tested along multiple axes such as geography, morphology and genetics. Recent studies such as Garg and Biju [23] also suggest that there might be several undescribed cryptic lineages within these genera.

Given this background, we used a large-scale spatial and taxon sampling design, which allowed us to identify sister lineages with confidence. We incorporated data from recent studies and tested for cryptic diversity in *Indirana* and *Walkerana* by comparing genetic and morphological divergence between closely related lineages. To do this, we first reconstructed a time-calibrated phylogeny of *Indirana* and *Walkerana* and derived genetic distances between sister lineages. We then determined levels of cryptic diversity by comparing genetic, geographical, and morphological distances between lineages. We hypothesized that members of the genera *Indirana* and *Walkerana* show limited to no morphological difference, irrespective of levels of genetic divergence and timing of diversification.

## Materials and methods

### Field and taxon sampling

**Fig 1:**
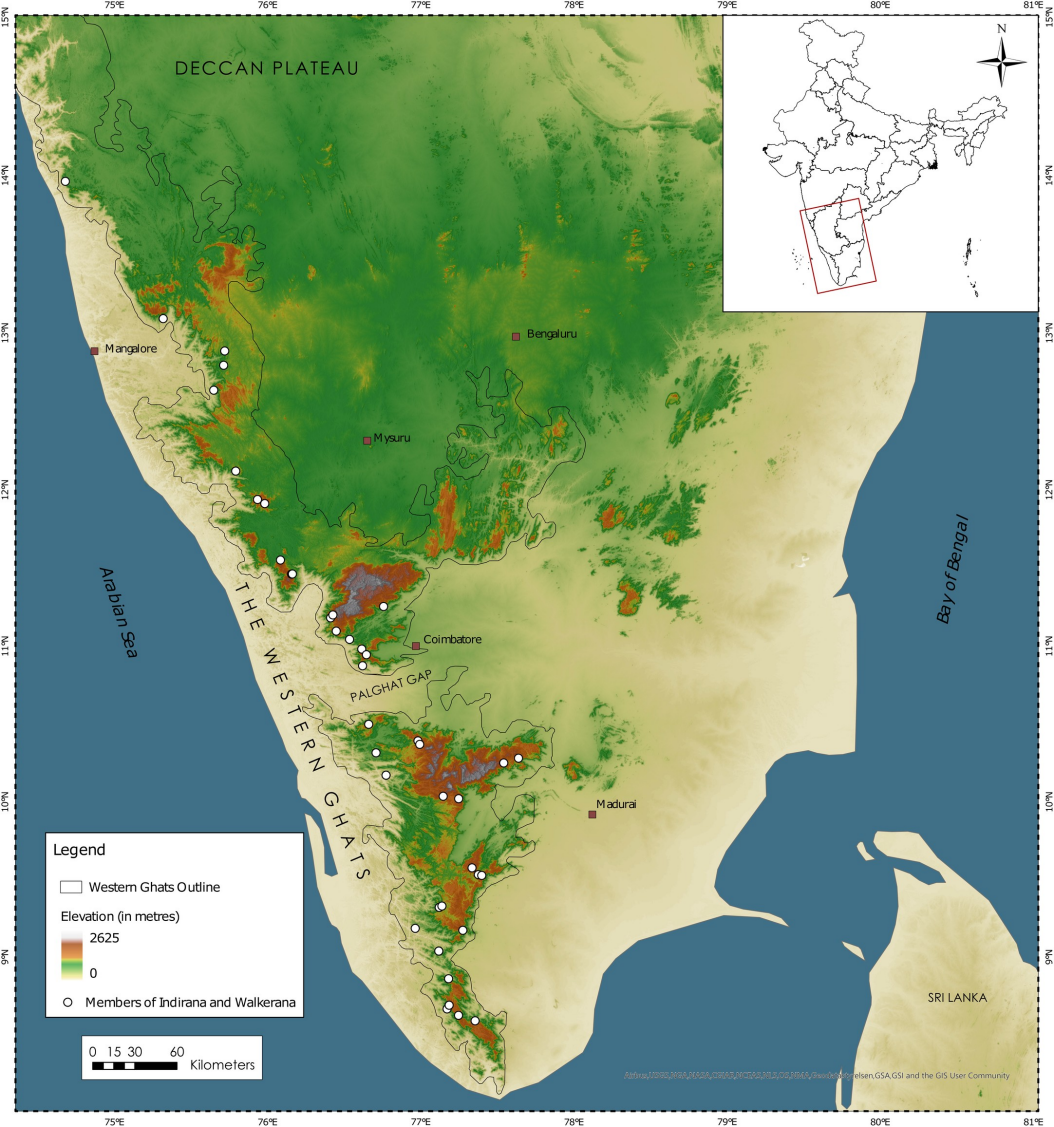
A map showing locations where individuals of *Indirana* and *Walkerana* were sampled over 2009–2013. This map was created using NaturalEarth Data in ArcGIS Pro.

The Western Ghats is a complex escarpment with greater topographic heterogeneity towards the southern regions with massifs that are over 2000 m high [25]. Individuals of *Indirana* spp. and *Walkerana* spp. were sampled during a series of herpetological expeditions as part of a larger project, which included collections from across all the major massifs in the Western Ghats along available elevational gradients and associated diversity of habitats (Fig 1). This spatial approach to sampling ensured that we captured the maximum variation (by sampling as many different populations as possible) and were able to assign sister lineages with confidence, a critical step for assessing cryptic variation. Specimens were collected during targeted sampling in rocky seepage areas, the typical habitat for many of these species and during chance encounters in forests, grassland, and human modified habitats. Collections were carried out across different seasons by various researchers over a period of five years (2009–2013). Specimens were euthanized by immersion in Tricaine Methanesulphonate (MS222) solution, fixed in 4% formalin or 70% Ethanol and preserved in 70% Ethanol. Tissue samples were stored in molecular grade 95% alcohol. For formalin fixed specimens, liver & thigh muscle tissues were extracted before fixation and stored in ethyl alcohol (95%). Morphological measurements were taken using Mitutoyo vernier calipers (to the nearest 0.1 mm).

### DNA extraction and sequencing

To augment existing DNA sequence data from published literature [20–23], we selected forty-four specimens of the genus *Indirana* and *Walkerana* in this study. Samples were selected in such a way that they represented potentially isolated populations across the entire range of the Western Ghats. Liver and muscle tissues were extracted and stored in absolute alcohol at -20°C. Extraction and isolation of DNA was performed using the standard phenol-chloroform method of extraction. The primers used by Simon et al. [26] were used to amplify the 16S mitochondrial gene. The PCR amplifications for 16S rRNA gene fragments were performed in 25ul reactions consisting of 2.5uL each of 10x PCR buffer, MgCl2 (25 mM) and deoxyribonucleotide triphosphates (2.5 mM), 0.25 uL of each primer (10 mM) 16Sar (Forward Primer): (5’-CGC CTG TTT ATC AAA AAC AT-3’) and 16Sbr (Reverse Primer): (5’-CTC CGG TTT GAA CTC AGA TCA-3’) and 0.67uL of Taq DNA polymerase (Amnion Biosciences), 15.33 μL of dH_2_O and 1uL of template DNA (25ng). The following thermocycling conditions were used: 94°C for 3 min, followed by 35 cycles of 94°C for 50 s, 46.8°C for 1 min, 72°C for 40 s, followed by a final extension step at 72°C for 5 min. The PCR products were purified using Qiagen purification kits© (Qiagen, New Delhi, India). The purified products were sequenced using 3130xl genetic analyzer, Applied Biosystems. Both the forward and reverse strands were sequenced for all 44 samples (GenBank Accession Numbers for specimens sequenced in this study: KX098602 –KX098645).

The DNA sequences obtained from individuals (n = 44) were aligned using MUSCLE in the R programming environment v 3.6.1 [27, 28]. To the above set of forty-four 16S sequences, we added available 16S sequences from recent studies [20–23]. The final alignment of 16S rRNA gene sequences was 483bp long and consisted of a total of 92 individuals which included all individuals of *Indirana* and *Walkerana* that were sequenced by Nair et al. [24], Padhye et al. [20], Modak et al. [21], Dahanukar et al. [22] and Garg and Biju [23]. This included the following species: *I*. *beddomii*, *I*. *semipalmata*, *W*. *diplosticta*, *I*. *brachytarsus* and *W*. *leptodactyla* (Genbank Accession Numbers: JQ596642-JQ596858) from Nair et al. [24]; *I*. *chiravasi* and *I*. *gundia* (Genbank Accession Numbers: KM386530-KM386533) from Padhye et al. [20]; *I*. *duboisi*, *I*. *tysoni*, *I*. *yadera* and *I*. *sarojamma* (Genbank Accession Numbers: KX641796, KX641815-KX641823, KX641858, KX641875-KX641882) from Dahanukar et al. [22]; *I*. *bhadrai* and *I*. *paramakri* (Genbank Accession Numbers: KX966036, KX966110-KX966112, KX966164, KX966170) from Garg and Biju [23]; and *I*. *salelkari* (Genbank Accession Numbers: KP826824-KP826826) from Modak et al. [21] (*see* S1 File) By matching all available published DNA sequences for *Indirana* and *Walkerana* with the DNA sequences generated in this study, we identified all extant species following Vijayakumar et al. [29].

### Phylogenetic analyses

**Fig 2:**
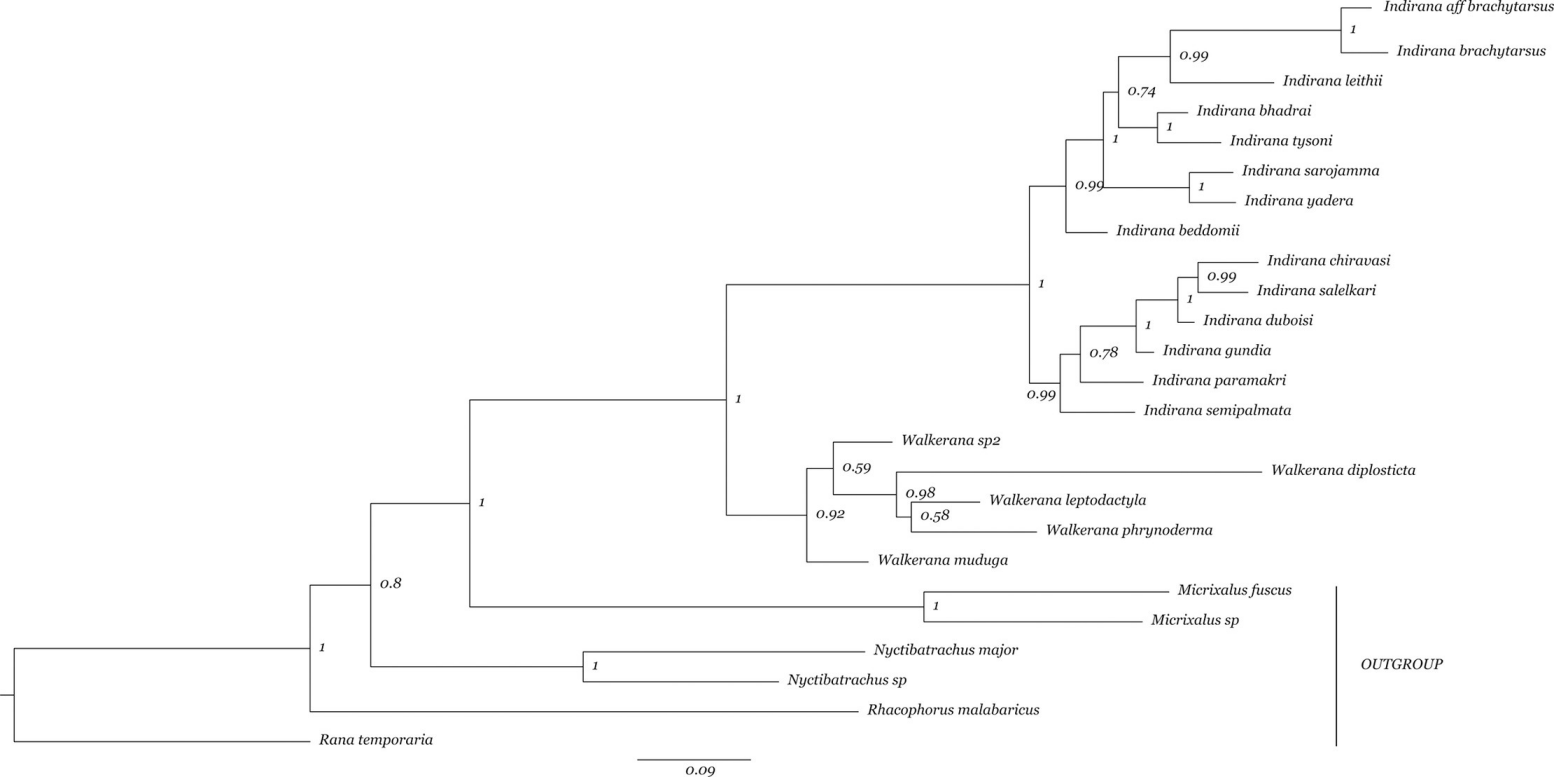
Bayesian species tree with posterior probability values indicated on the nodes. Majority of the clades had high support (>0.9) except for a few unresolved nodes.

Before phylogenetic analyses was carried out, we incorporated published DNA sequence data for two mtDNA genes (12S and CO1) and two nuclear genes (Rag1 and Rhodopsin) and our 16S gene data to create a concatenated multigene dataset. This concatenated multigene dataset consisted of data from five genes and was used to carry out a Bayesian phylogenetic analysis as described below. The final alignment consisted of a total of 112 individuals (of which 25 individuals were used to construct a species tree, *see* Fig 2), which included 92 individuals of *Indirana* and *Walkerana* and others from the families Micrixalidae, Nyctibatrachidae and Sooglossidae which were used as outgroups [30].

Bayesian phylogenetic analysis was performed using MrBayes v3.2.2 on the concatenated dataset [31]. We used PartitionFinder 2 to identify the best models of sequence evolution along with partitions, by using a greedy algorithm with linked branch lengths and Bayesian information criteria for the concatenated dataset [32] (*see* S1 File) The dataset was independently run twice for 20 million generations with a random starting tree and four markov chains (three hot and one cold), sampling every 2000th generation. Adequate burn-in was determined using a plot of the likelihood scores of the heated chain for convergence in TRACER v1.6 [33]. The trees obtained were visualized in FIGTREE v 1.4.3 and R programming environment v 3.6.1 [28, 34].

### Divergence dating

Divergence dating analyses was performed using BEAST v1.8.2 [35]. The concatenated multi-gene dataset, consisting of all 112 individuals was used to estimate divergence times. Node calibrations for members of the outgroup were based on Feng et al. [30] (*see* S1 File). Since the node calibrations are secondary calibrations derived from Feng et al. [30], we used a normal distribution with 95% confidence intervals (*see* S1 File). We used an uncorrelated relaxed lognormal clock across all partitions, with a Birth-Death speciation tree prior and an uninformative prior for all partition rates. Two independent analyses were run for 100,000,000 generations, sampling every 10000 generations. Convergence was determined using TRACER v1.6, based on ESS values [33]. A burn-in of 25% was used and a maximum clade credibility tree (MCC) was obtained using TREEANNOTATOR v 1.8.2 [36]. The final trees were visualized using FIGTREE v 1.4.3 [34].

### Species delimitation

We used a hierarchical multi-criteria approach to delimit lineages, following Vijayakumar et al. [29]. First, we constructed a haplotype tree with the combined 16S dataset (published DNA sequences and the DNA sequences generated in this study), using a Maximum Likelihood approach in MEGA7 [37]. We identified haplotype clusters (>70% bootstrap support) using the 16S haplotype tree and verified this with the multigene tree generated previously. We then calculated pairwise genetic distances between these lineages on the 16S gene [38]. Wherever phylogenetic resolution precluded identifying the sister lineage or the closest relative, pairwise genetic distances were calculated for all other lineages and the minimum distance was taken into consideration for further analyses. We classified lineages as shallow (< 3%), moderate (3% to 4%) and highly (> 4%) divergent lineages based on their pairwise genetic distances on the 16S rRNA gene (following Vijayakumar et al. [29] and Fouquet et al. [39]) While certain genetic distance values have been previously used as thresholds or cutoffs to delimit amphibian species (Fouquet et al. [39]), our goal here was to test for cryptic diversity across varying levels of genetic divergence. Hence, we only used genetic distance to bin lineages into different classes of divergence for further analysis. These lineages were then examined in multivariate morphological space to identify cryptic lineages.

### Identifying cryptic lineages

We define cryptic lineages as those that overlap in morphological space despite moderate to high levels of genetic divergence. Morphological similarity is anticipated in the case of shallow divergent lineages, but lineages that were more genetically divergent were expected to show some degree of morphological divergence. In order to determine if individuals of the genus *Indirana* and *Walkerana* form distinct morphological groups with respect to levels of genetic divergence, we conducted nonmetric multidimensional scaling (nMDS) to summarize underlying trends in the morphometric parameters, and used the partitioning around medoids (PAM) technique to detect the presence of distinct morphometric groups in multivariate space. We also conducted simple Mantel’s tests to examine the relationship between geographic distance, genetics and morphometry.

The dataset included morphometric measurements of 10 variables for 88 frogs of the genera *Indirana* and *Walkerana*. This final dataset of 10 morphometric variables was derived from a larger dataset of 34 morphological variables by combining data from this study and from Dahanukar et al. [22], and accounting for correlations among them [40]. Phylogenetic analyses (described in the section above) revealed the presence of 19 lineages (*see* Results), which were genetically and geographically distinct. However, morphometric data was available for only 15 of the 19 lineages identified. The final morphometric dataset consisted of measurements that included head diameter (HD), head length (HL), eye diameter (ED), eye to snout distance (ES), upper eyelid width (UEW), interorbital width (IO), internarial distance (IN), upper arm length (UAL), lower arm length (LAL), and horizontal tympanum diameter (TYH). All the above measurements were standardized with respect to Snout Vent Length (SVL) for each individual.

nMDS is an ordination technique in which trends in underlying data are mapped to a reduced ordination space, such that the distances between samples in ordination space reflects the overall morphological differences between individuals. Considering the correlations among the morphometric variables, we calculated the Mahalonobis distance matrix using the ecodist statistical package in the R programming environment v 3.6.1 [28, 41]. To determine the number of axes to be used in the nMDS ordination, we ran the first round of ordinations with six dimensions using 10 iterations. We then extracted the stress values generated and determined the minimum, maximum and mean stress values and plotted them against the number of dimensions to generate a scree plot. These stress values indicate the fit of the data in ordination space where the lower the stress, the greater the fit of the data to the model.

Similarly, we determined the explanatory power for 1–6 dimensions (*see* S1 File). Based on the explanatory power and stress values for 1–6 dimensions, a final analysis was conducted with 2 dimensions, using 20 iterations. Lastly, to describe the ordination axes, we correlated the morphometric variables with the ordination axes and plotted the same using the vector fitting method. nMDS plots were plotted for individuals that belong only to the genera *Indirana* and *Walkerana* to test for similarity in morphometric parameters, irrespective of phylogenetic relationships or levels of genetic divergence (2% to 11%).

Using the statistical package Cluster in R, we used the partitioning around medoids (PAM) technique to detect the presence of distinct groups in multivariate space [42]. Partitioning around medoids is one of the pooling or K-means clustering algorithms included as a non-hierarchical agglomeration classification technique. The algorithm uses ‘medoids’ as the centroid of the pool, which is the exemplar point of the cluster of points in ordination space. Silhouette widths were initially plotted against a range of 2–10 clusters to determine the number of clusters to be chosen for final analysis. Each silhouette represents a cluster of samples indicating which sample lies within the cluster (*see* S1 File). The clusters were then plotted in ordination space to check for sorting based on morphometric measurements.

Lastly, using the ecodist statistical package, we conducted simple mantel’s correlations between the Mahalanobis distances of morphometric variables and the geographical and genetic distances between pairs of individuals [41]. A permutation approach was used to test significance (10000 permutations). All analyses were performed in the R programming environment v 3.6.1 [28].

## Results

### Phylogenetic analysis

Of the 17 extant species that have been described within the *Indirana* and *Walkerana* genera today, our taxon sampling captured 88.2% of species (15 of 17 species), with the exception of *I*. *bhadrai* and *I*. *salelkari* as shown in the species tree (Fig 2). Phylogenetic analysis also revealed the presence of three potential lineages, of which one lineage belonged to *Indirana* (*Indirana* aff. *brachytarsus*) and two belonged to *Walkerana* (*Walkerana muduga* which was described recently [43]; and *Walkerana* sp2) (Fig 2). Phylogenetic analysis based on multiple genes supports the sister relationship of the two clades, *Indirana* and *Walkerana* (Fig 2). Most of the sister-lineage pairs were well supported (p>0.9).

### Divergence dating

**Fig 3:**
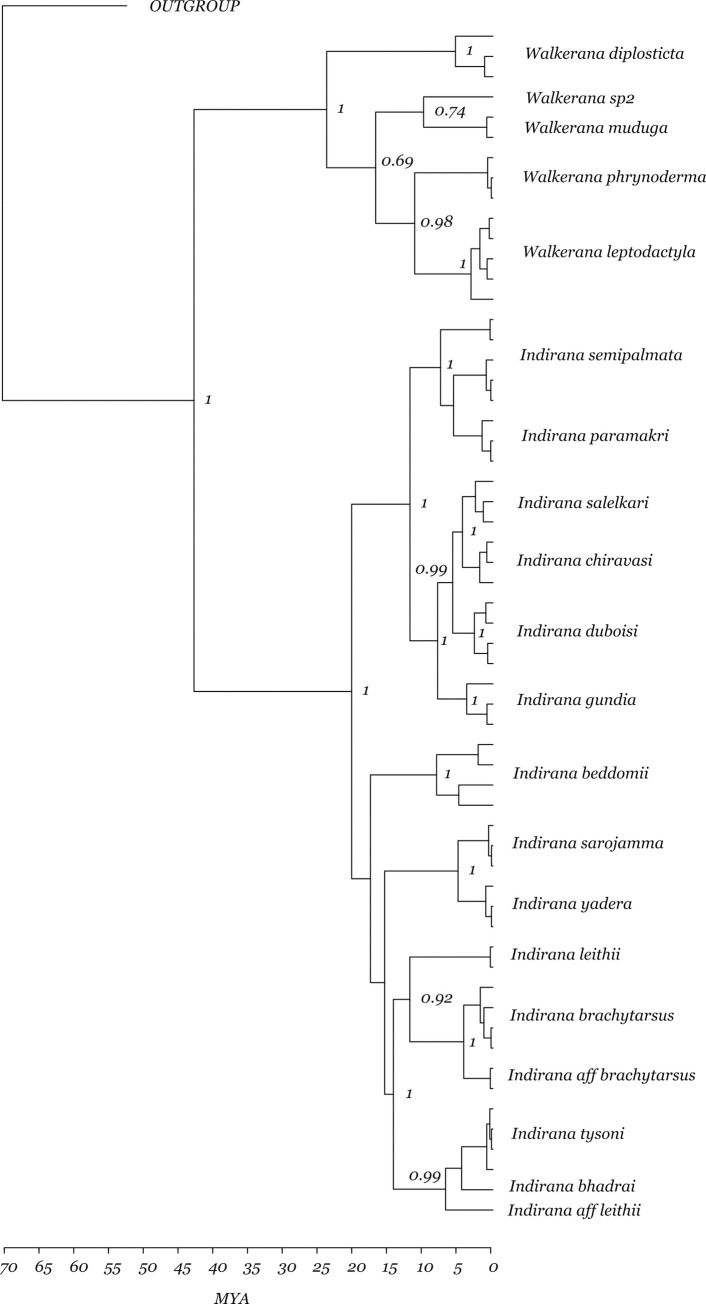
A time calibrated phylogeny of *Indirana* and *Walkerana* with divergence estimates in millions of years indicated.

Each of the individual BEAST runs converged (ESS > 200). A chronogram with posterior probability values is reported (Fig 3). The clade comprising *Indirana* and *Walkerana* began diversifying around 71 mya (53 mya– 88 mya) and the most recent common ancestor of *Indirana* and *Walkerana* split around 43 mya (31 mya– 58 mya). However, diversification within *Indirana* and *Walkerana* began around the same time ie. 24 mya (15 mya– 35 mya) and 20 mya (14 mya– 27 mya) for the two genera, respectively (*see* S1 Fig for divergence estimates for the outgroup and S2 Fig for median ages (95% HPD)).

### Testing for levels of cryptic diversity

**Fig 4:**
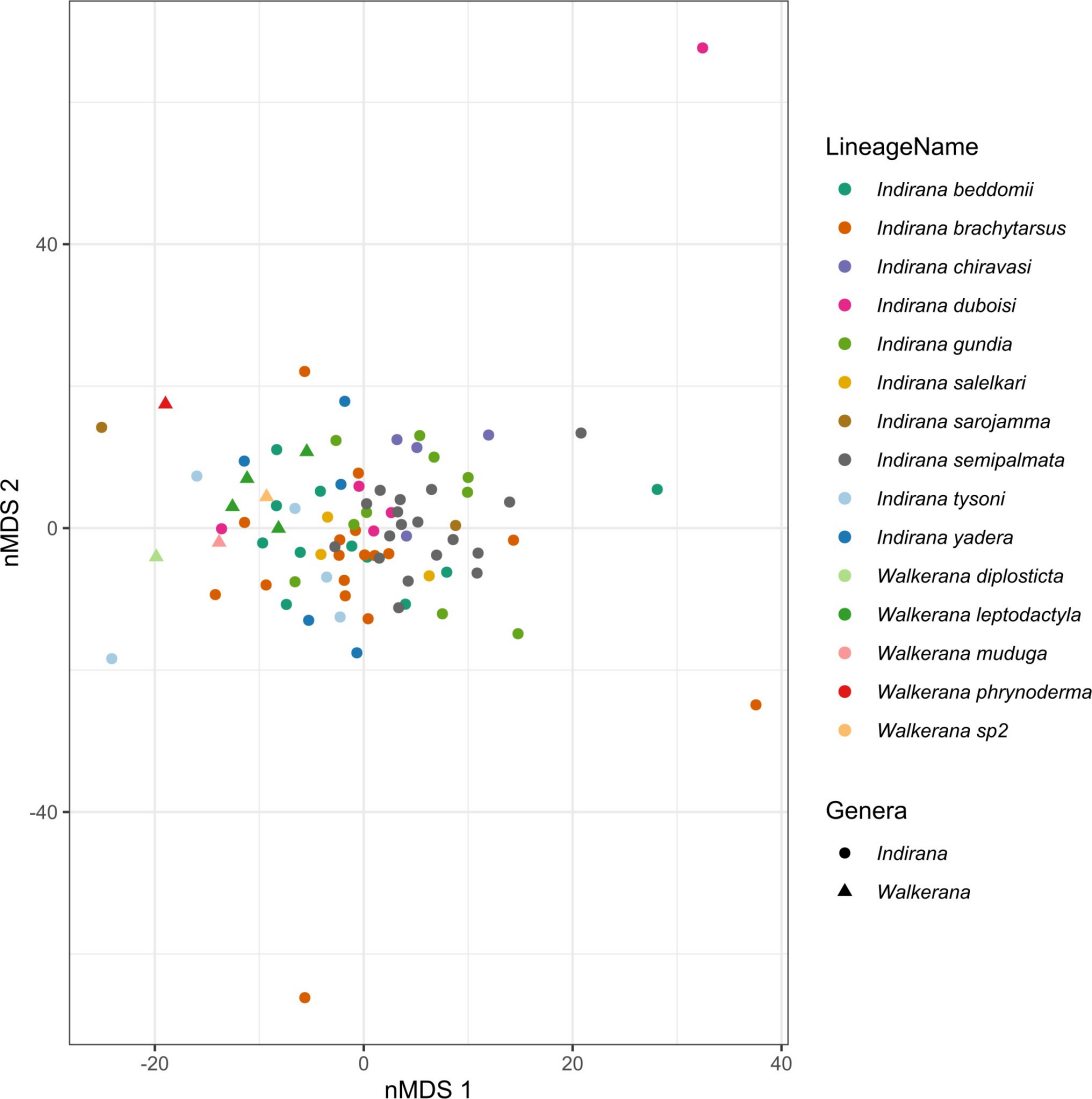
Nonmetric Multidimensional Scaling (nMDS) ordination representing the sampled frogs colored by lineages. nMDS axis 1 explains 55.08% of the variability in morphological composition and nMDS axis 2 explains 28.14%. The ordination suggests that irrespective of their genetic distance, the lineages clustered in morphological space. Refer to lineage numbers in S1 File.

nMDS analyses revealed overlap of sister lineages with similar and indistinguishable morphometry, indicated by the points representing the morphometric measurements in multivariate morphological space. Hence, members of the genus *Indirana* and *Walkerana* were found to be cryptic. nMDS analyses resulted in two axes that explained 83.23% of the variability in the morphology of the 15 lineages included. nMDS axis 1 explained 55.08% of the variability in morphological composition and nMDS axis 2 explained 28.14% (Fig 4). Head diameter (HD), eye diameter (ED), horizontal tympanum diameter (TYH) and Interorbital width (IO) were positively correlated and had the highest loading on nMDS axis 1 and upper eyelid width (UEW) and internarial distance (IN) were negatively correlated with nMDS axis 1 (p<0.05). Horizontal tympanum diameter (TYH) was positively correlated and had the highest loading on nMDS axis 2 (p<0.05) (*see* S3 Fig).

The sister-pairs which differed from one another with varying degrees of genetic distances (2–11% on 16S gene) showed strong overlap in morphological space. In addition, there was significant overlap in morphological space for all species of the genus *Indirana* and all species of the genus *Walkerana* (S4 and S5 Figs). In the clustering analysis, the silhouette width plot indicated a sharp increase for three clusters. On plotting the result of the PAM algorithm for 3 clusters, we qualitatively concluded the absence of distinct clusters as indicated by the silhouette plot being close to zero, as was the case for 10 clusters. Furthermore, on plotting the 10 clusters in ordination space, it is visually clear that the clusters do not sort or separate in any manner (S6 Fig). Hence, we inferred that irrespective of the number of clusters used, there are no distinct clusters formed and the structure is weak.

Simple Mantel’s correlations found no relationship between morphometric parameters and geographic distance (Mantel’s r = 0.002, p = 0.92) and between morphometric parameters and genetic distance (p-distances) (Mantel’s r = 0.00, p = 0.91) However, genetic (p-distances) and geographic distance were positively correlated (Mantel’s r = 0.195, p = 0.0001).

## Discussion

Cryptic species, or lineages that are genetically divergent but exhibit morphological similarity, pose a taxonomic challenge for biodiversity and conservation [2]. With increasing rates of species discovery, we need a unified framework for identifying taxa which not only considers the evolutionary processes resulting in cryptic species, but can estimate occurrences of such taxa across varying environments to inform conservation [1, 5]. For example, Mayr and Ashlock [44] suggested that one could infer a degree of difference in what they called ‘good species’ i.e. those that are morphologically separated from their sister species, to identify those that are cryptic. Others such as Funk et al. [45] have advocated the use of mating calls to distinguish between species that are deemed morphologically cryptic, but this is only possible in species that vocalize.

Shanker et al. [46] proposed the extension of the generalized lineage concept (de Queiroz [47]) and provide a framework for the use of multiple axes such as phylogenetics, morphology and geography for species delimitation. However, the literature on cryptic species delimitation is fraught with opposing points of view on how one should define ‘cryptic species’ [48]. Irrespective of the uncertainty revolving around the species concept, it is important that we incorporate multiple criteria to identify such lineages for the conservation of biodiversity [49, 50]. In this study, we highlight the use of multiple axes to detect high levels of cryptic diversity within the amphibian genera *Indirana* and *Walkerana* from the Western Ghats.

A total of 14 lineages were identified in this study within the genus *Indirana* and 5 lineages within *Walkerana*. The Bayesian concatenated gene tree was well resolved with high posterior probability values of >0.9, except for a few members of *Walkerana*. The Bayesian analyses recovered multiple lineages that were absent in recent work, such as *Indirana* aff. *brachytarsus*, *Walkerana muduga* (which was described recently [43]) and *Walkerana* sp2. The potential lineages, especially those that are geographically isolated, can be treated as a potential species, whose validity must be ascertained along multiple axes. For example, *Indirana* aff. *brachytarsus* and its sister *Indirana brachytarsus* differ by a genetic distance of 2% on the 16S gene. However, there is a lack of morphological and geographical data. On the other hand, our study identified two highly divergent lineages belonging to the genus *Walkerana* (*Walkerana muduga* [43] and *Walkerana* sp2) divergent by 3.8% on the 16S gene from each other and exhibiting deep genetic divergence from extant lineages (3.8% to 13.5% on the 16S gene).

The nMDS analysis suggests discordance between genetic and morphological divergence. Sister lineages did not show signs of morphological separation in multivariate space irrespective of their level of genetic divergence. Similar patterns were seen across most of the lineages, except for *Walkerana phrynoderma*, a morphologically distinct and highly divergent lineage restricted to the Anamalai massif (Fig 4). Other lineages sampled at high elevations such as *Walkerana muduga* (Dinesh et al. [43]) and *Walkerana sp2* did not show signs of morphological separation in the nMDS analysis. This lack of morphological divergence among cryptic taxa has been reported in other studies from South East Asia and the Neotropics. For instance, Stuart et al. [51] found that sympatric lineages of ranid frogs in Southeast Asia were morphologically cryptic, and Fouquet et al. [39] similarly found cryptic lineages of shallow divergence in sympatry within the neotropical frog genera, *Scinax* and *Rhinella*.

The geological and climatic history of this region has provided multiple opportunities for diversification and speciation of morphologically distinct lineages [52]. A majority of the diversification within these genera occurred during the Miocene (~24 mya and ~20 mya for *Indirana* and *Walkerana* respectively), coinciding with a period of shift in vegetation along with the intensification of monsoon seasonality [53, 54]. Hence, the creation of an ecological gradient along with geographic isolation could have led to the allopatric speciation of ancient morphologically distinct lineages such as *Walkerana phyrnoderma* [55]. Bush frogs (*Raorchestes*) in the Western Ghats show considerable morphological diversification through adaptation to different habitats [29]. Given that *Indirana/Walkerana* habits/niches may be largely similar, perhaps morphological stasis has been favored. Thus, lowland lineages such as *Indirana semipalmata* may have remained morphologically cryptic due to an inability to exploit novel niches or as a result of decoupling between lineage and morphological diversification, as shown in *Hemidactylus* geckos [54].

The detection of cryptic species suggests that divergence along the axes of genetics and morphology are not necessarily correlated [46]. A recent review proposed mechanisms surrounding cryptic species diversification, one of which is the ‘recent divergence’ hypothesis, which posits that cryptic taxa have diverged recently and hence, morphological differentiation is not evident as such traits evolve slowly over time [49, 56]. The presence of cryptic diversity in *Indirana* and *Walkerana* is surprising given the age of the clade and the potential opportunities for diversification in this heterogeneous landscape. Given that cryptic diversity has been detected in evolutionarily old taxa such as these suggests other mechanisms such as phylogenetic niche conservatism i.e. the tendency for certain lineages to retain their ecological niches over time [56, 57]. Evidence for this is not restricted to amphibians alone in this region, as ancient lineages of geckos have been reported to be cryptic [58]. Lastly, morphological similarity or convergence could also be a result of similar selection pressures [59].

The presence of cryptic lineages across a range of divergence levels in Ranixalids demonstrates a strong case of morphological stasis within the clade and highlights a unique model to understand historical drivers of morphological diversity. Due to the underlying problem of identifying *Indirana* and *Walkerana* in the field, extensive taxon sampling in space would be needed to define range limits for the cryptic species observed here. While the use of geography can be useful in testing the presence of cryptic lineages, the challenge of defining the geographical range of a cryptic taxon remains. Our study is also based on a limited number of morphological variables and it is important to test for possibilities of divergence along unexamined axis of morphology such as shape, webbing etc. Divergence along additional axes such as advertisement calls needs to be tested for their utility in identifying morphologically cryptic lineages in the field.

There are many species-rich endemic and ancient clades of comparable age to *Indirana* and *Walkerana* in the Western Ghats, such as *Nyctibatrachus* and *Micrixalus* [19]. Considering their underlying biological differences, these are potential models to understand the historical drivers of cryptic diversity and to identify variables of significance in discerning species boundaries in tropical hotspots such as the Western Ghats. Exploration along ecological and geographic axes, along with robust sampling can potentially assist in parsing these drivers of cryptic diversification.

## Supporting information

S1 File (XLSX)

S2 File (R)

S1 Fig Time calibrated phylogeny of the outgroup used for divergence dating analysis. (PNG)

S2 Fig Time calibrated phylogeny of the Ranixalidae clade with 95% confidence intervals around median ages, depicted on the nodes. (PNG)

S3 Fig Biplot indicating the composition of the ordinate axis in terms of the morphometric measurements. (PNG)

S4 Fig Nonmetric multidimensional scaling (nMDS) ordination representing sister-pairs. Shown here are all lineages belonging to the genus *Indirana*. Refer to lineage numbers in S1 File.
(PNG)

S5 Fig Nonmetric multidimensional scaling (nMDS) ordination representing sister-pairs. Shown here are all lineages belonging to the genus *Walkerana*. Refer to lineage numbers in S1 File.
(PNG)

S6 Fig Partitioning Around Medoids (PAM) plot with 10 groups indicating no distinct groups in ordination space. (PNG)
