# Supplementary figures and images for: Determining levels of cryptic diversity within the endemic frog genera, Indirana and Walkerana, of the Western Ghats, India
Source: PLoS One. 2020 Sep 2;15(9):e0237431. doi: 10.1371/journal.pone.0237431 (PMC7467320; doi:10.1371/journal.pone.0237431)

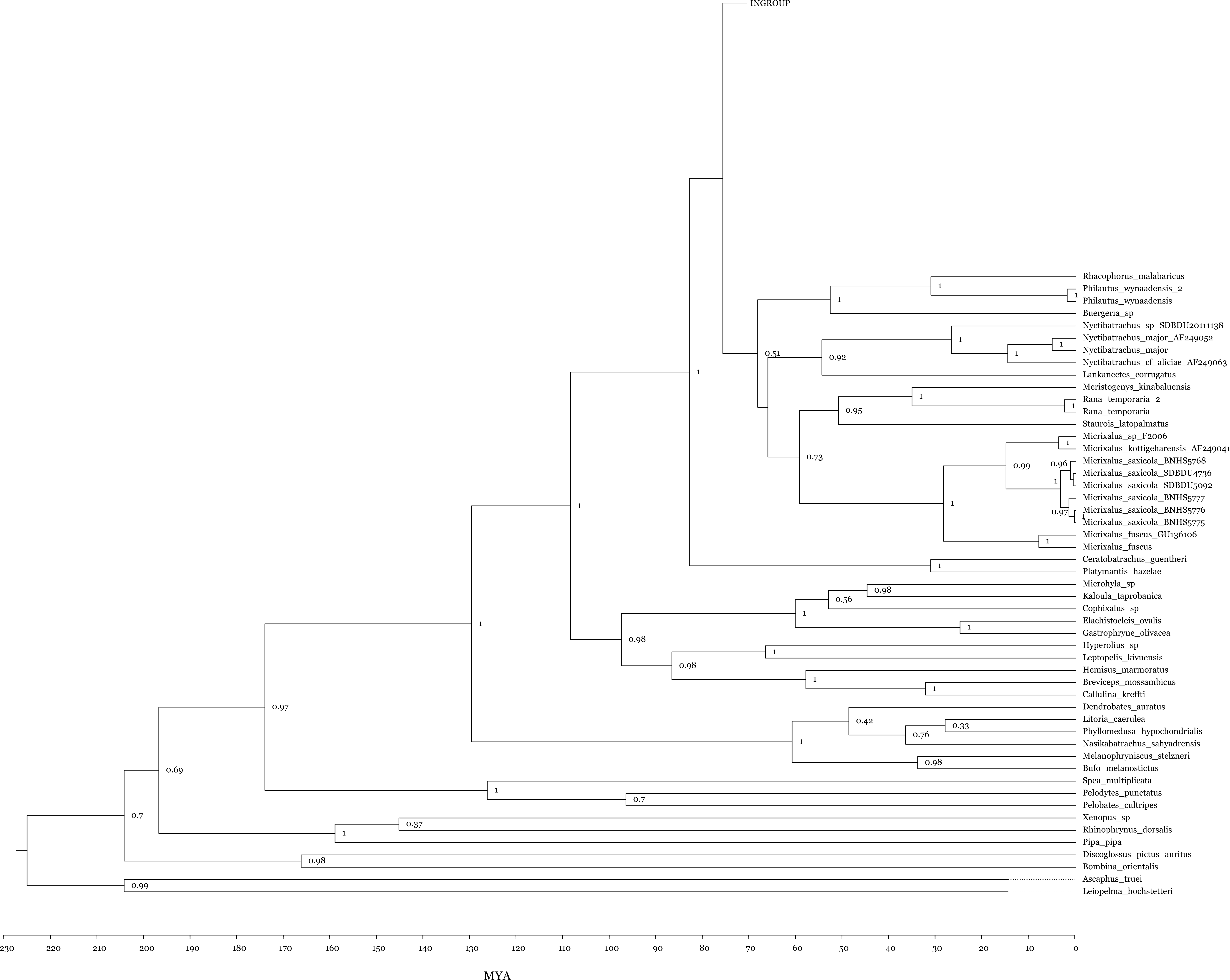

Supplement: S1 Fig — (PNG) [file pone.0237431.s003.png]

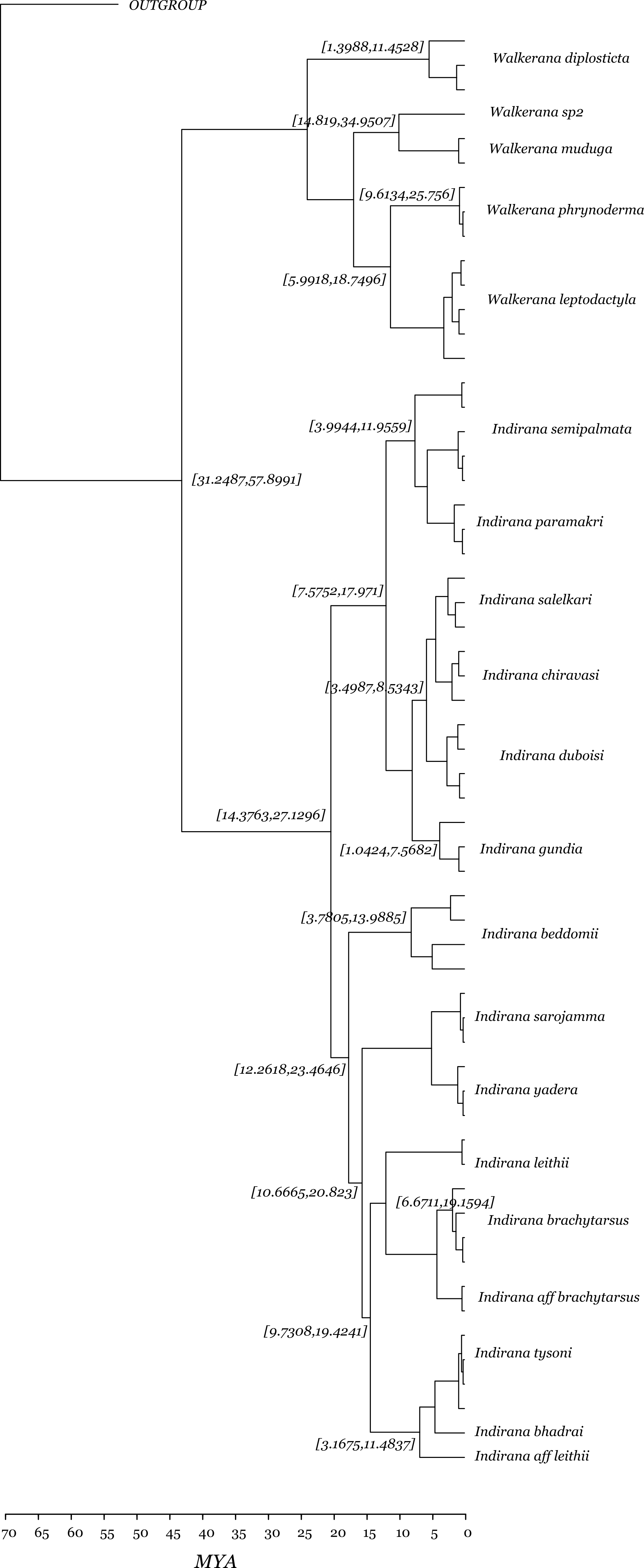

Supplement: S2 Fig — (PNG) [file pone.0237431.s004.png]

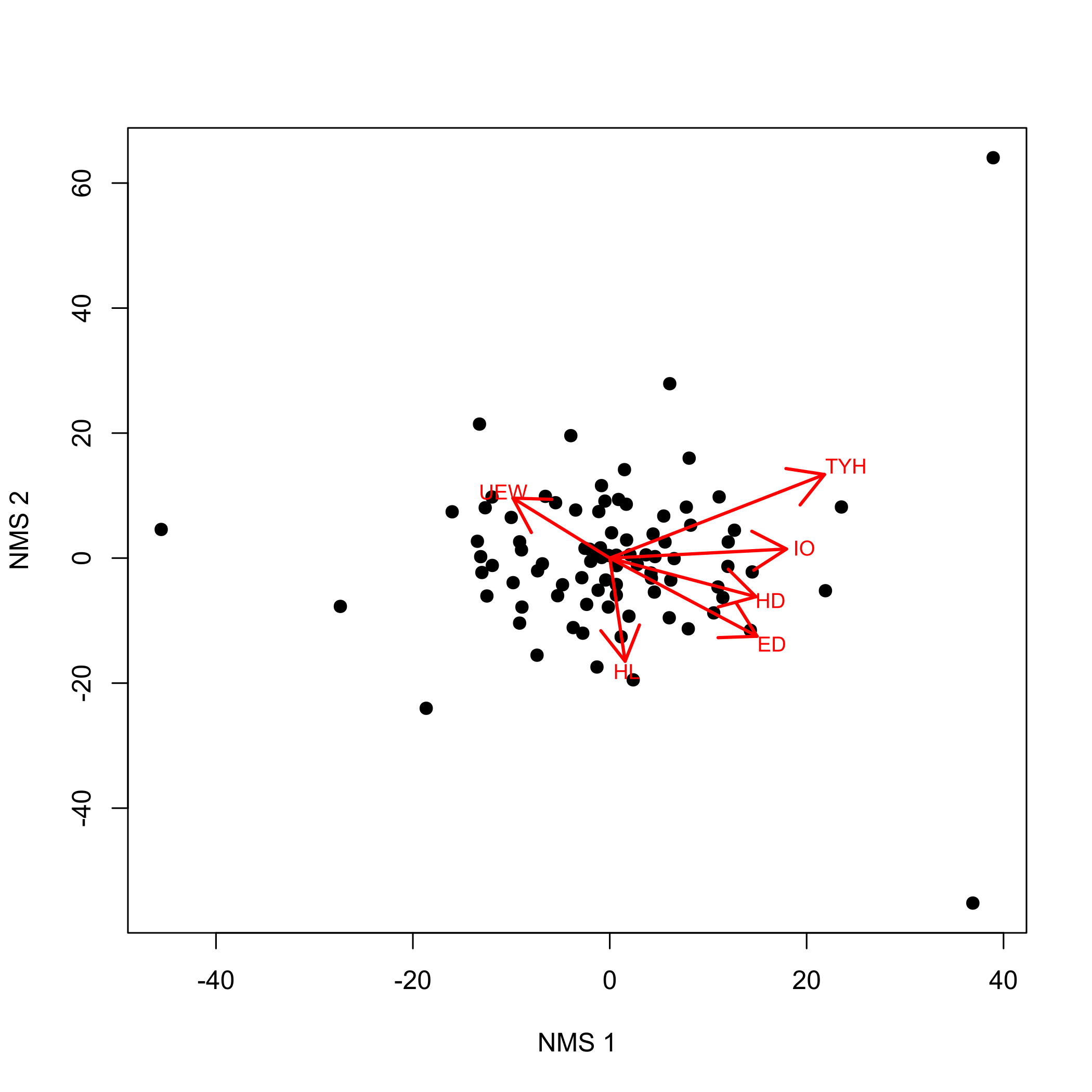

Supplement: S3 Fig — (PNG) [file pone.0237431.s005.png]

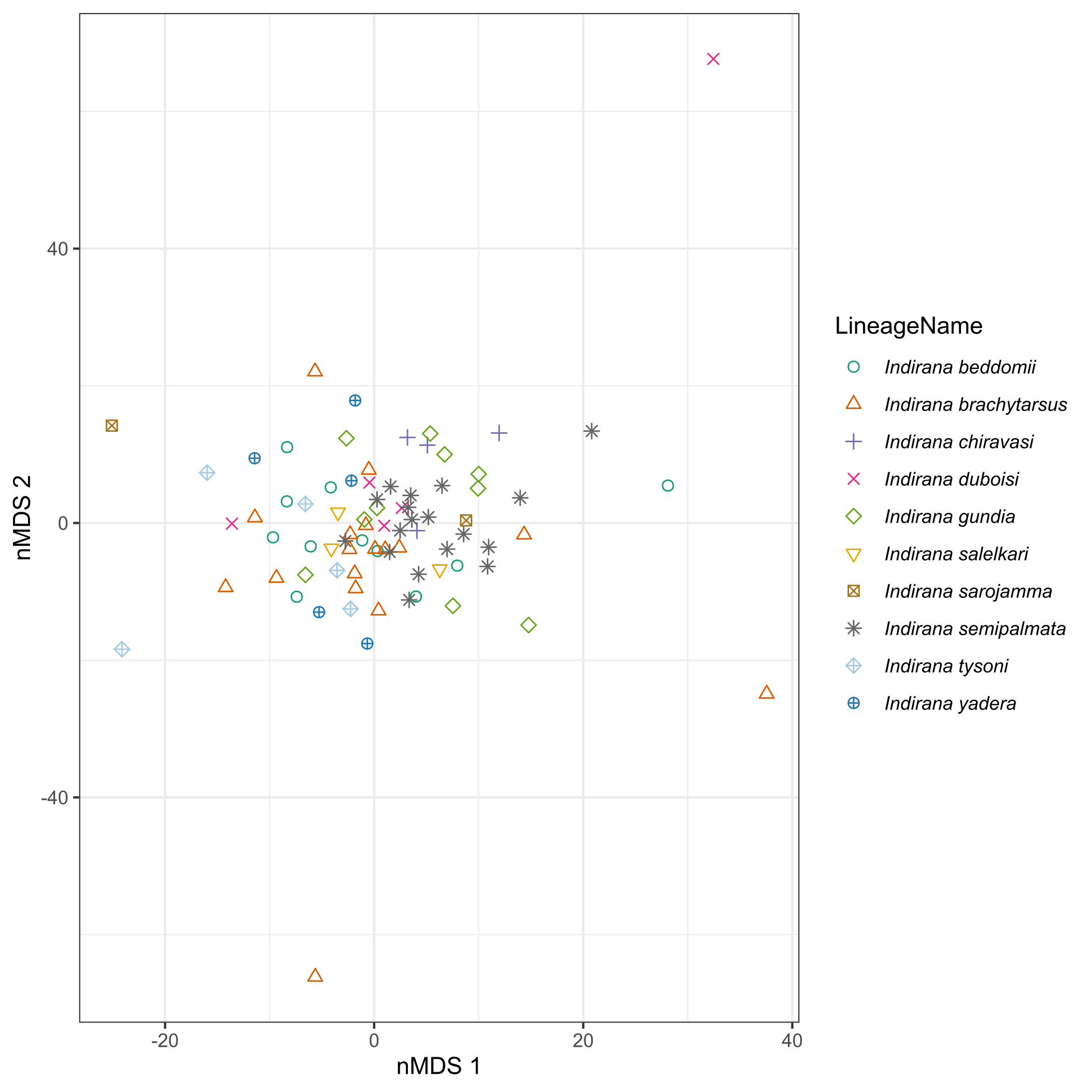

Supplement: S4 Fig — Shown here are all lineages belonging to the genus Indirana. Refer to lineage numbers in S1 File. (PNG) [file pone.0237431.s006.png]

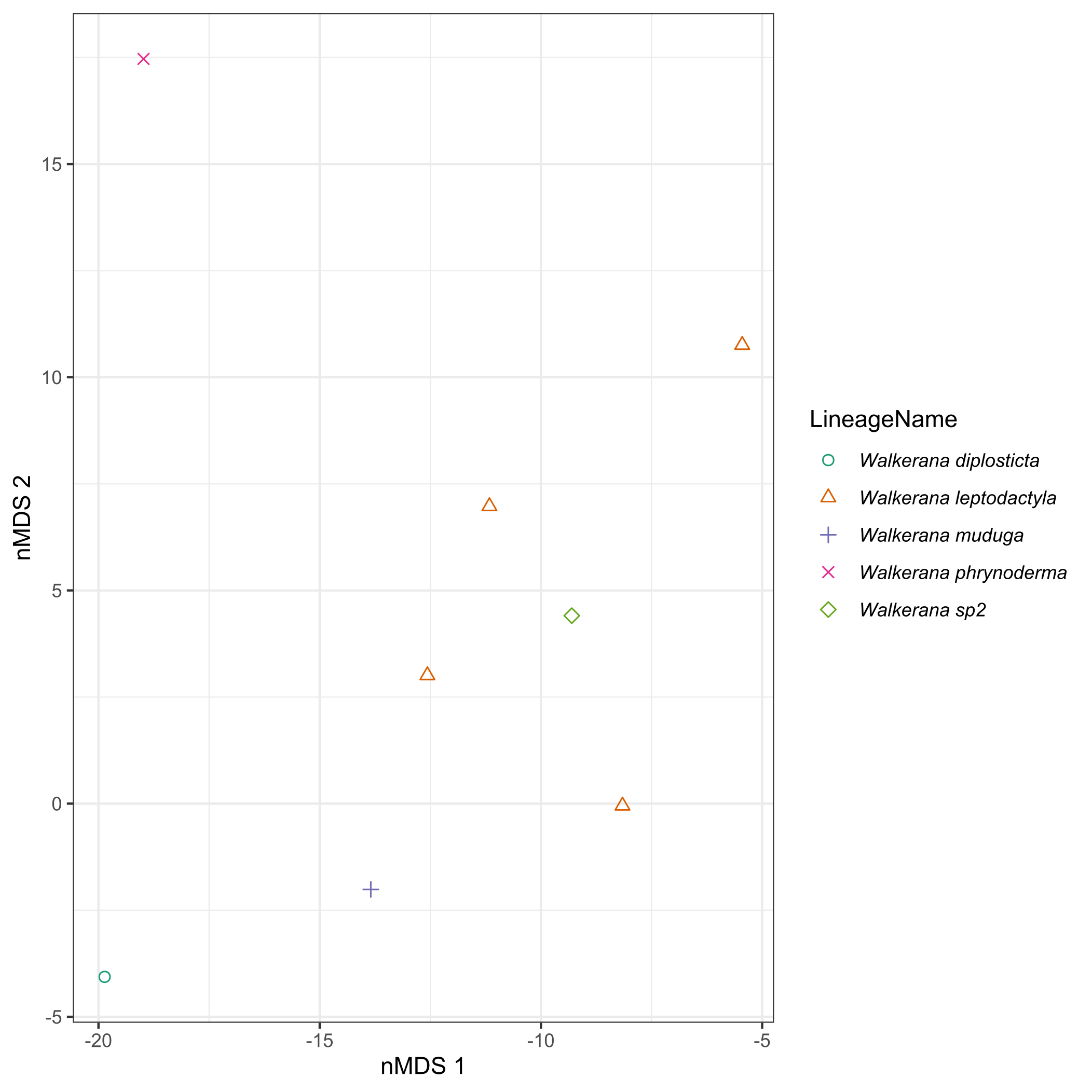

Supplement: S5 Fig — Shown here are all lineages belonging to the genus Walkerana. Refer to lineage numbers in S1 File. (PNG) [file pone.0237431.s007.png]

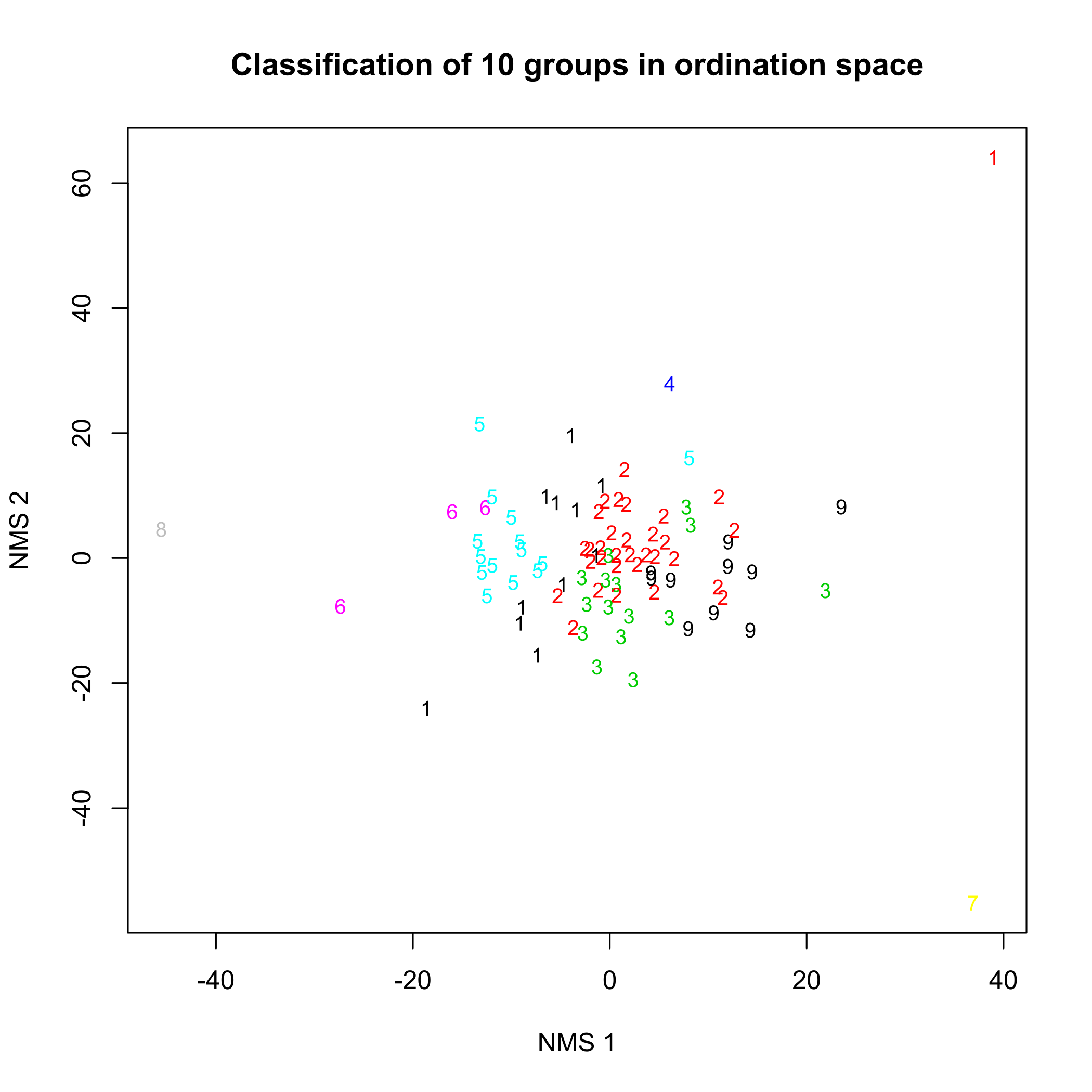

Supplement: S6 Fig — (PNG) [file pone.0237431.s008.png]
